# Activation and detection of HTLV-I Tax-specific CTLs by Epitope expressing Single-Chain Trimers of MHC Class I in a rat model

**DOI:** 10.1186/1742-4690-5-90

**Published:** 2008-10-08

**Authors:** Takashi Ohashi, Mika Nagai, Hiroyuki Okada, Ryo Takayanagi, Hisatoshi Shida

**Affiliations:** 1 Division of Molecular Virology, Institute for Genetic Medicine, Hokkaido University, Sapporo, 060-0815, Japan

## Abstract

**Background:**

Human T cell leukemia virus type I (HTLV-I) causes adult T-cell leukemia (ATL) in infected individuals after a long incubation period. Immunological studies have suggested that insufficient host T cell response to HTLV-I is a potential risk factor for ATL. To understand the relationship between host T cell response and HTLV-I pathogenesis in a rat model system, we have developed an activation and detection system of HTLV-I Tax-specific cytotoxic T lymphocytes (CTLs) by Epitope expressing Single-Chain Trimers (SCTs) of MHC Class I.

**Results:**

We have established expression vectors which encode SCTs of rat MHC-I (RT1.A^l^) with Tax180-188 peptide. Human cell lines transfected with the established expression vectors were able to induce IFN-γ and TNF-α production by a Tax180-188-specific CTL line, 4O1/C8. We have further fused the C-terminus of SCTs to EGFP and established cells expressing SCT-EGFP fusion protein on the surface. By co-cultivating the cells with 4O1/C8, we have confirmed that the epitope-specific CTLs acquired SCT-EGFP fusion proteins and that these EGFP-possessed CTLs were detectable by flow cytometric analysis.

**Conclusion:**

We have generated a SCT of rat MHC-I linked to Tax epitope peptide, which can be applicable for the induction of Tax-specific CTLs in rat model systems of HTLV-I infection. We have also established a detection system of Tax-specific CTLs by using cells expressing SCTs fused with EGFP. These systems will be useful tools in understanding the role of HTLV-I specific CTLs in HTLV-I pathogenesis.

## Background

Human T-cell leukemia virus type I (HTLV-I) is etiologically linked to adult T-cell leukemia (ATL) [1,2], a chronic progressive neurological disorder termed HTLV-I-associated myelopathy/tropical spastic paraparesis (HAM/TSP) [3,4], and various other human diseases [5-8]. ATL is a malignant lymphoproliferative disease affecting a subgroup of middle-aged HTLV-I carriers characterized by the presence of mature T cell phenotype [9]. HTLV-I genome contains a unique 3' region, designated as pX, which encodes the viral transactivator protein, Tax [10]. Because of its broad transactivation capabilities [11], it is speculated that Tax plays a central role in HTLV-I associated immortalization and transformation of T cells, which may lead to the development of ATL.

Tax is also known as a major target protein recognized by cytotoxic T lymphocytes (CTL) of HTLV-I carriers [12]. It has been reported that the levels of HTLV-I-specific CTL are quite diverse among HTLV-I carriers and that ATL patients have impaired levels of HTLV-I specific CTLs in contrast to the high levels of CTL response in HTLV-I carriers with HAM/TSP [13-15]. In addition, it has been known that HTLV-I Tax-specific CTL response was strongly activated in ATL patients who acquired complete remission after hematopoietic stem cell transplantation [16]. Based on these observations, it is speculated that HTLV-I-specific immune response may contribute to repressing the growth of HTLV-I infected cells in the infected individuals and insufficient host T cell response against HTLV-I may be a risk factor for ATL.

To understand the mechanism of ATL development, it is very important to dissect the interplay between the virus-specific CTLs and HTLV-I infected T cells. We have previously established a rat model of ATL-like disease, which allows examination of the growth and spread of HTLV-I infected cells, as well assessment of the effects of immune T cells on the development of the disease [17,18]. By using this model system, we also reported the therapeutic effect of Tax-coding DNA or peptide against the disease [19,20]. For further analyzing the effects of Tax specific CTLs in the rat model, it is important to develop effective methods to activate Tax specific CTLs and to detect the virus-specific CTLs.

It has been reported that single chain trimers (SCTs) of MHC-I have the potential to efficiently stimulate and identify antigen specific T cells in both human and mouse systems [21,22]. In this system, all three components of MHC-I complexes, such as an antigen peptide, β_2_-microgrobulin (β_2_m), and MHC-I heavy chain are covalently attached with flexible linkers. By linking together the three components into a single chain chimeric protein, a complicated cellular machinery of normal antigen processing can be bypassed, leading to stable cell surface expression of MHC-I coupled with an antigenic peptide of interest. In addition, a new system has been established to identify virus-specific T cells using the acquisition mechanism of epitope/MHC complex by CD8 T cells through MHC/TCR interaction [23].

In this study, to establish an activation system of Tax-specific CTLs in our rat model system, we have generated a SCT of rat MHC-I linked to Tax epitope peptide. We have also established a detection system of Tax-specific CTLs by using cells expressing SCTs fused with EGFP. These newly established systems would be useful tools in understanding the role of HTLV-I specific CTLs in HTLV-I pathogenesis.

## Results

### Production and functional capabilities of peptide-β_2_m-RT1.A^l ^fusion proteins

**Figure 1:**
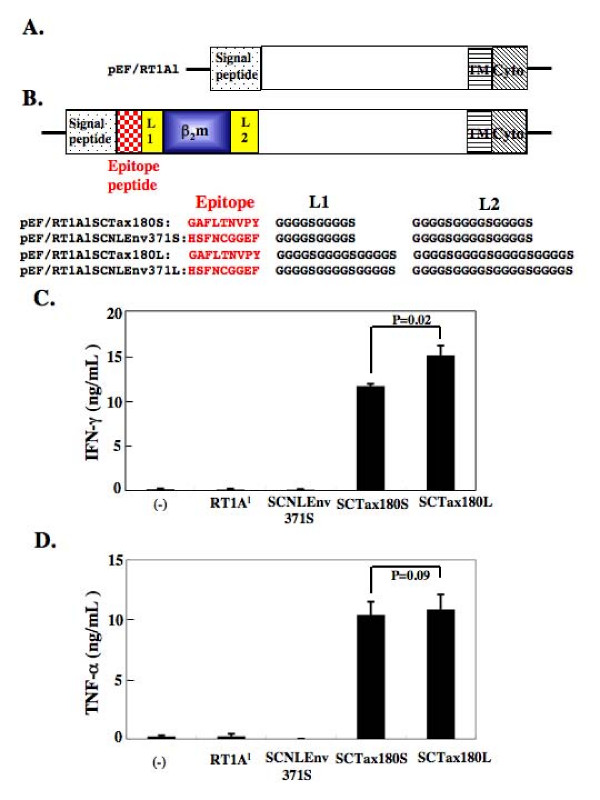
**Activation of Tax-specific CTLs by 293T cells expressing SCTs with Tax 180–188 epitope.** (A) Diagram of full-length rat MHC-I (RT1.A^l^). (B) Diagram of SCTs encoding Tax180-188 or NLEnv371-379 linked to β_2_m and RT1.A^l ^molecules with different lengths of linkers. L1, linker 1; TM, transmembrane region; Cyto, cytoplasmic region. (C and D) The 293T cells were either untreated or transfected with pEF/RT1Al, pEF/RT1AlSCNLEnv371S, pEF/RT1AlSCTax180S, or pEF/RT1AlSCTax180L. The 293T cells were then incubated with a Tax-specific CD8+ T cell line, 4O1/C8. Production of IFN-γ (C) and TNF-α (D) in the supernatants was measured by ELISA after 24 hours of culture. The data represent the mean ± the SD of triplicate wells. Similar results were obtained in two independent experiments.

To establish an activation system of Tax-specific CTLs using SCTs of rat MHC-I (RT1.A^l^), we have constructed expression vectors as illustrated in Figure 1A. Tax180-188 epitope was previously identified as an RT1.A^l^-restricted CTL epitope recognized by a Tax-specific CTL line [20]. As a negative control in this study, we have chosen a putative RT1.A^l^-restricted epitope in the envelope of HIV-1 NL4-3 strain, NLEnv371-379, which was determined to have the same point as the Tax180-188 epitope scored by epitope prediction data via [24]. Since the linker length has been reported to influence the immune detection of SCTs in a mouse system [21], we have prepared SCTs with Tax180-188 or NLEnv 371–379 peptide linked by different lengths of spacers. We then performed an in vitro transfection experiment to assess the effects of SCTs for the activation of Tax-specific CTLs. The 293T cells were transfected with pEF/RT1Al, pEF/RT1AlSCNLEnv371S, pEF/RT1AlSCTax180S, or pEF/RT1AlSCTax180L. These transfected 293T cells were subsequently used to stimulate an RT1.A^l^-restricted HTLV-I Tax180-188-specific CTL line, 4O1/C8. As shown in Figure 1B, 293T/RT1AlSCTax180S and 293T/RT1AlSCTax180L cells were able to induce IFN-γ secretion by 4O1/C8. Statistical analysis revealed a significant increase of IFN-γ production (P = 0.02) in 293T/RT1AlSCTax180L cells compared with 293T/RT1AlSCTax180S. In contrast, 293T/RT1Al, 293T/RT1AlSCNLEnv371S, and nontransfected 293T cells induced little IFN-γ secretion by the Tax-specific CTLs. We have also confirmed the induction of TNF-α production by these vectors, although there was no significant difference observed between 293T/RT1AlSCTax180L and 293T/RT1AlSCTax180S cells (Figure 1D). These results suggested that Tax180-188/β_2_m/RT1.A^l ^SCTs were efficiently expressed on the cell surface of the transfected cells and were recognized by the epitope-specific CTLs.

### Establishment of MOLT-4 cells stably expressing SCTs of RT1.A^l^

**Figure 2:**
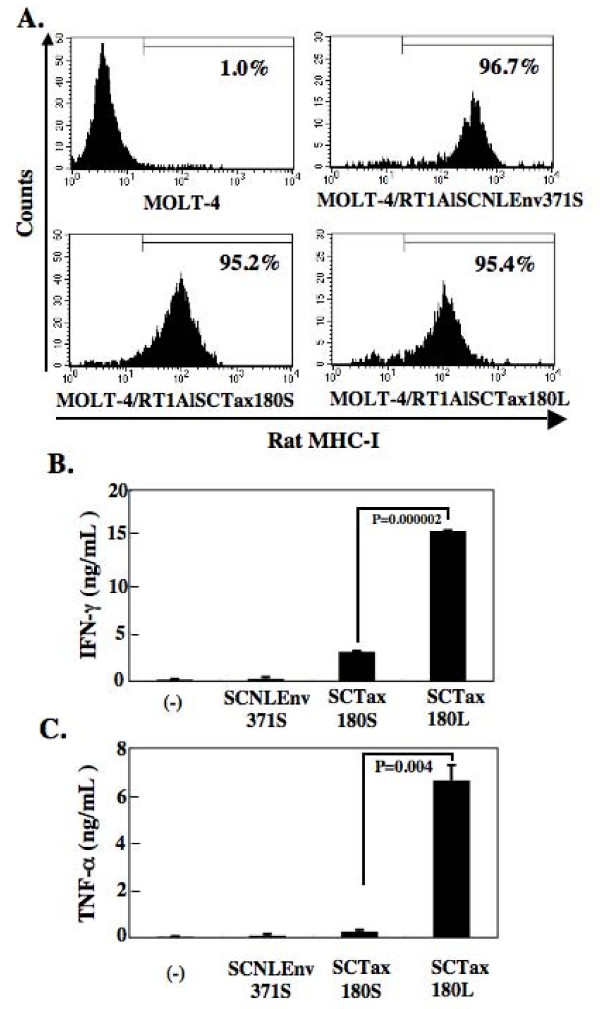
**Establishment of MOLT-4 cells stably expressing SCTs of RT1.A^l^.** (A) MOLT-4 cells were transfected with various SCT expression vectors. After selection by G418 and cloning, flow cytometric analysis was performed to determine the expression level of RT1.A^l ^on MOLT-4 cells. The percentage of RT1.A^l^-positive cells is indicated in each part. (B and C) The MOLT-4 cells expression with indicated SCTs were incubated with a Tax-specific CD8+ T cell line, 4O1/C8. Production of IFN-γ (B) and TNF-α (C) in the supernatants was then measured by ELISA after 24 hours of culture. The data represent the mean ± the SD of triplicate wells. Similar results were obtained in two independent experiments.

To examine the effects of rat SCTs expressed on human cells and the influence of linker length on the activation of CTLs in more detail, we have introduced the expression vectors into MOLT-4 cells and established the cells stably expressing SCTs of RT1.A^l ^with the different linker length. After selection by G418 and cloning, FACS analysis was performed to determine the expression level of RT1.A^l ^on MOLT-4 cells. As shown in Figure 2A, equivalent levels of SCT expression were confirmed on the surface of MOLT-4/RT1AlSCTax180S and MOLT-4/RT1AlSCTax180L cells, whereas we detected higher mean fluorescence intensity (MFI) in MOLT-4/RT1AlSCNLEnv371S compared with the other 2 SCT-transfected cells. These SCTs expressing MOLT-4 cells were subsequently used to stimulate 4O1/C8 cells. As shown in Figure 2B and 2C, MOLT-4/RT1AlSCTax180S and MOLT-4/RT1AlSCTax180L cells were able to induce both IFN-γ and TNF-α secretions by 4O1/C8. MOLT-4/RT1AlSCTax180L induced significantly higher levels of IFN-γ and TNF-α than those induced by MOLT-4/RT1AlSCTax180S, suggesting that the SCT with the longer linker has a higher affinity to the epitope-specific TCR. In contrast, MOLT-4/RT1AlSCNLEnv371S cells induced little IFN-γ and TNF-α secretion by the Tax-specific CTLs, despite the higher expression of SCTs. Parental MOLT-4 cells did not stimulate the cytokine secretion, either. These results indicated that the SCTs with longer linkers have the advantage to efficiently stimulate the epitope-specific CTLs and suggested that the longer form would be suitable for further application of immunological study.

### Inhibitory effects of SCTs expressing Tax180-188 on the growth of Tax-specific CTLs

**Figure 3:**
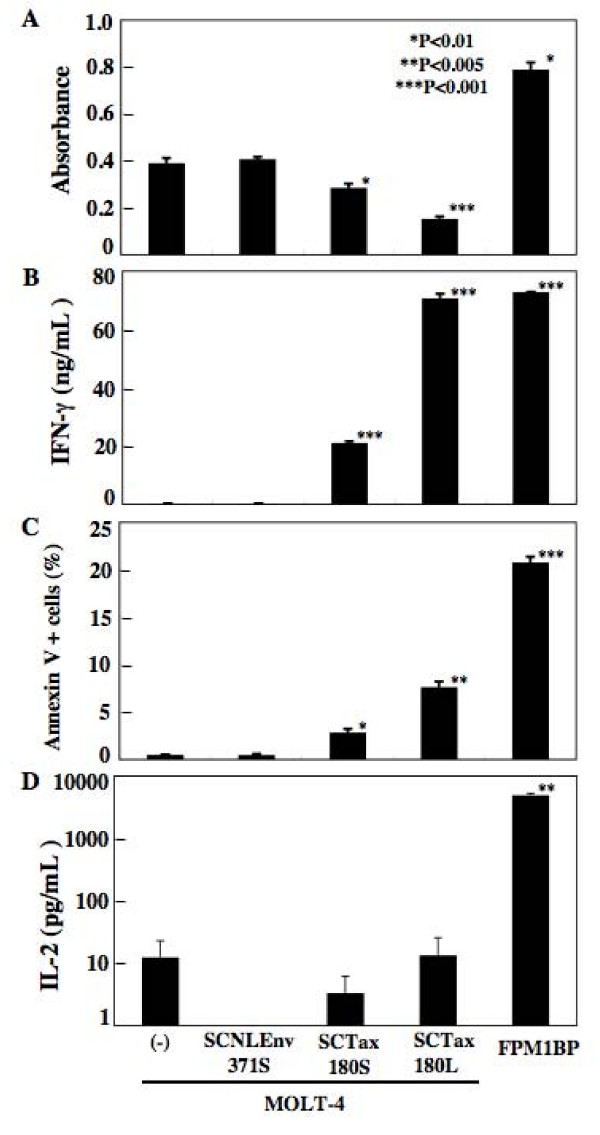
**(A) Inhibitory effects of SCTs expressing Tax180-188 on the growth of Tax-specific CTLs.** An HTLV-I infected syngeneic rat cell line, FPM1.BP or various MOLT-4 cells were fixed with formalin and were then mixed with 4O1/C8. After 3 days of mixed culture, the growth of 4O1/C8 was evaluated using cell counting kit-8. (B) Production of IFN-γ in the culture supernatants was measured by ELISA after 3 days of mixed culture. (C) Apoptotic status of 4O1/C8 was evaluated by staining with Annexin V-FITC and anti-rat CD8 Ab-PE. (D) Production of IL-2 in the culture supernatants was measured by ELISA after 2 days of mixed culture. *P < 0.01, **P < 0.05, and ***P < 0.001 compared to the mixed culture with parental MOLT-4 cells. The data represent the mean ± the SD of triplicate wells. Similar results were obtained in two independent experiments.

We next examined whether the SCTs could induce the expansion of epitope-specific CTLs in vitro. A series of SCT-expressing MOLT-4 cell lines were fixed with formalin and then used as stimulators for 4O1/C8. An HTLV-I infected syngeneic rat cell line, FPM1.BP, was also used as a stimulator, because it has been used to stimulate 4O1/C8 cells and was thus known to induce the proliferation of the CTLs. After 3 days of mixed culture, the growth of 4O1/C8 was evaluated. As shown in Figure 3A, FPM1.BP cells significantly enhanced the growth of 4O1/C8 as compared with untransfected MOLT-4 cells. In contrast, MOLT-4 cells expressing SCTs with Tax180 did not induce the proliferation of 4O1/C8, but significantly inhibited the growth of the CTLs. We detected stronger growth inhibition in MOLT-4 cells with longer linkers than those with shorter linkers. The expression of SCTs with NLEnv371 on MOLT-4 cells caused no influence on the growth of 4O1/C8. We also assessed the IFN-γ production in the mixed culture and confirmed the significantly high level of the cytokine in the culture of FPM1.BP. It was of note that IFN-γ production was inversely correlated with the growth of 4O1/C8 among the mixed cultures of MOLT-4 cells with different SCTs, suggesting that observed growth inhibition was due to the activation induced cell death (AICD). Thus, we further investigated the apoptotic status of 4O1/C8 by Annexin V staining. As shown in Figure 3C, we observed the increase of Annexin V positive cells after mixed culture with MOLT-4 cells expressing SCTs with Tax180, but not with those expressing SCTs with NLEnv371S. As correlated with the growth inhibition, the SCTs with longer linker induced higher rate of apoptosis in 4O1/C8 cells than those with shorter linker did. It is of note that a much higher level of apoptosis was observed in the mixed culture of FPM1.BP cells, indicating that FPM1.BP was able to promote the growth of 4O1/C8 even though it induced a higher level of AICD at the same time. To understand the mechanism of enhanced proliferation induced by FPM1.BP, we have assessed the IL-2 concentration in the mixed culture and found that production of the T cell-stimulatory cytokine was dramatically enhanced by FPM1.BP cells (Figure 3D). These results suggested that the growth inhibition by SCTs with Tax resulted from both an enhanced level of AICD and a reduced activation of proliferation signal(s) including IL-2 pathways, which FPM1.BP cells were able to stimulate.

### Detection of Tax-specific CTLs by SCTs fused with EGFP

**Figure 4:**
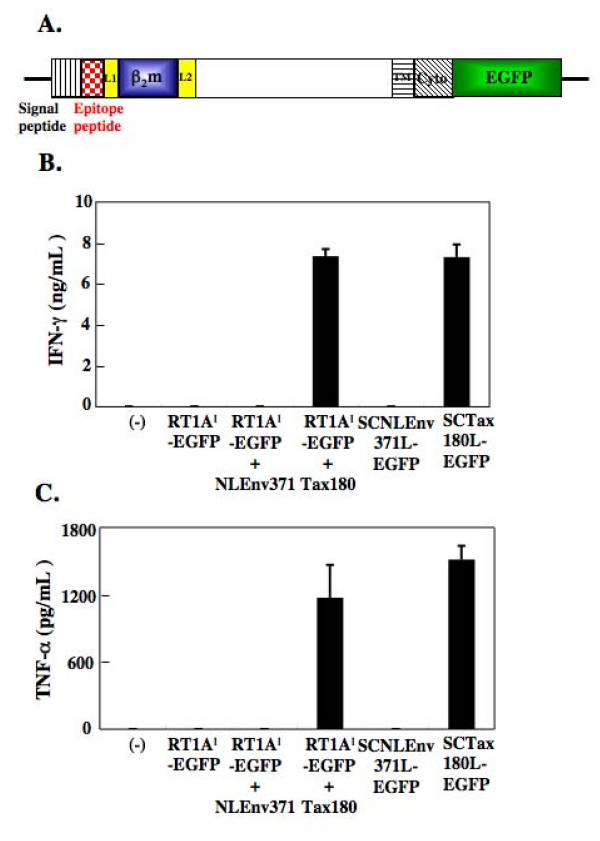
**Expression of SCTs of RT1.A^l ^fused with EGFP. (A) Diagram of SCTs of RT1.A^l ^fused at its C-terminal end to EGFP.** (B and C) The 293T cells were either untreated or transfected with pEF/RT1Al-EGFP, pEF/RT1AlSCNLEnv371L-EGFP, or pEF/RT1AlSCTax180L-EGFP. After 48 hours of transfection, the 293T cells were incubated with 4O1/C8 cells for 24 hours. Production of IFN-γ (B) and TNF-α (C) in the supernatants was measured by ELISA. For 293T/RT1Al-EGFP cells, NLEnv371-379 or Tax180-188 peptides were pulsed for 30 min before the mixed culture with 4O1/C8. The data represent the mean ± the SD of triplicate wells. Similar results were obtained in two independent experiments.

To establish a detection system of Tax-specific CTLs, the single chain peptide-RT1.A^l ^construct was then fused at its C-terminal end to EGFP as illustrated in Figure 4A. We have prepared two constructs with covalently linked Tax180-188 or NLEnv371-379 peptides with longer linkers, which were designated as pEF/RT1AlSCTax180L-EGFP and pEF/RT1AlSCNLEnv371L-EGFP, respectively. We have also generated a construct, which can express only RT1.A^l ^fused at its C-terminus to EGFP (pEF/RT1Al-EGFP). These vectors were transfected into 293T cells to express fusion proteins on the surface. To determine whether SCTs with EGFP are properly expressed on the surface of 293T cells, we have incubated the transfected 293T cells with 4O1/C8 and then assessed the IFN-γ and TNF-α production in the mixed culture. As shown in Figure 4B and 4C, neither 4O1/C8 cells mixed with parental 293T nor those with 293T/RT1Al-EGFP produced detectable levels of IFN-γ and TNF-α. When we pulsed the 293T/RT1Al-EGFP with 10 μM of Tax180-188 peptides, but not with NLEnv371-379 peptides, for 30 min before co-cultivation, we clearly detected the increase of IFN-γ and TNF-α production in the culture. The 293T cells expressing RT1AlSCTax180L-EGFP also induced IFN-γ and TNF-α production, but those expressing RT1AlSCNLEnv371L-EGFP did not. These results indicated that RT1.A^l^-EGFP fusion proteins with epitope peptides were efficiently recognized by Tax-specific CTLs.

**Figure 5:**
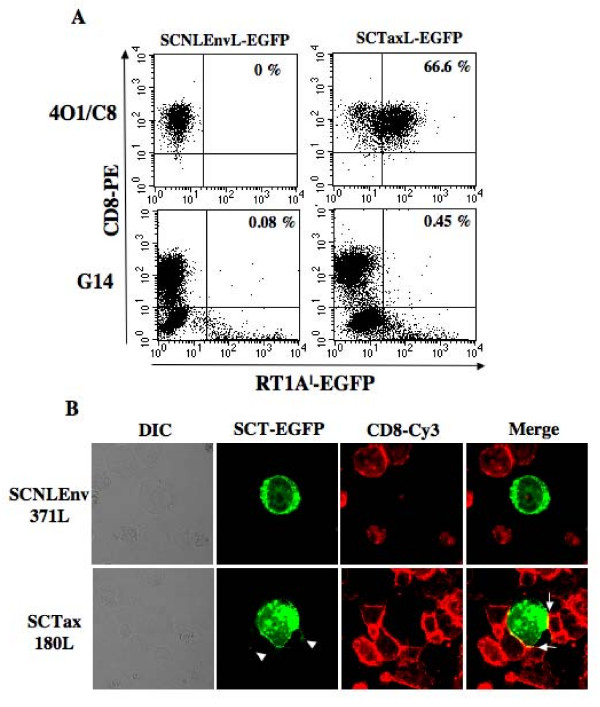
**Detection of Tax-specific CTLs by SCTs fused with EGFP. (A) The 293T cells transfected with pEF/RT1AlSCNLEnv371L-EGFP or pEF/RT1AlSCTax180L-EGFP were incubated with 4O1/C8 or control G14 cells.** After 1 hour of mixed culture, cells were stained with PE-conjugated anti-rat CD8 antibody and EGFP expression on CD8+ cells were assessed by flow cytometric analysis. (B) Cells in the mixed culture of 4O1/C8 and EGFP-expressing 293T cells were attached on slide glasses by centrifugation, fixed with 4% paraformaldehyde for 15 min at room temperature and then stained with an anti-rat CD8 antibody in combination with a Cy3-conjugated goat anti-mouse IgG (H+L) antibody. Fluorescence and differential interference contrast (DIC) images were obtained with a confocal microscope system and a pair of GFP and CD8 images was overlaid (merge). Arrowheads indicate SCT-EGFP in 4O1/C8 cells. Arrows indicate co-localization of SCT-EGFP and CD8 at the contact site. Similar results were obtained in two independent experiments.

To determine whether SCTs with EGFP can be acquired by antigen-specific CTLs, we incubated the transfected 293T cells together with 4O1/C8 cells or another CD8+ syngeneic T cell line, G14, which is not specific to Tax 180-188. As shown in Figure 5A, more than 60% of 4O1/C8 cells appeared to be positive for EGFP after mixed culture with 293T/RT1AlSCTax180L-EGFP cells for 1 hour, but not with 293T/RT1AlSCNLEnv371L-EGFP. In contrast, we were unable to detect G14 cells acquiring EGFP after mixed culture with 293T/RT1AlSCTax180L-EGFP. To confirm the acquisition of SCT-EGFP fusion proteins by 4O1/C8, we examined the cells by confocal microscopy. As shown in Figure 5B, SCT-EGFP with Tax180 molecules formed large clusters at 4O1/C8-293T contact sites (arrows) and appeared in 4O1/C8 cells (arrowheads) after 1 hour of mixed culture. In contrast, we were unable to detect the acquisition of EGFP fusion proteins by the CTLs after the mixed culture with 293T/RT1AlSCNLEnv371L-EGFP. Thus, RT1.AlSCTaxL-EGFP fusion proteins were specifically acquired by the epitope specific CTLs.

### Detection of Tax-specific CTLs in splenocytes derived from HTLV-I infected rats

**Figure 6:**
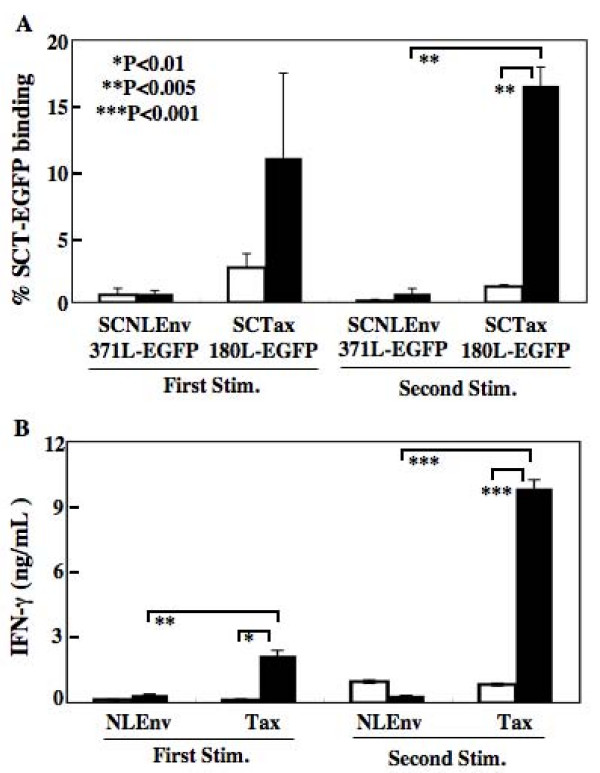
**Detection of Tax-specific CTLs in primary splenocytes stimulated with FPM1.BP cells in vitro (A) Splenocytes were isolated from an HTLV-I infected (▪) or uninfected control (□) rat and then stimulated with formalin-fixed FPM1.BP cells twice with 1-week interval.** One week after the first or second stimulation, splenocytes were purified and then incubated with the 293T cells transfected with pEF/RT1AlSCNLEnv371L-EGFP or pEF/RT1AlSCTax180L-EGFP. One hour after the mixed culture, cells were stained with PE-conjugated anti-rat CD8 antibody and EGFP expression on CD8+ cells were assessed by flow cytometric analysis. The percent CD8+ cells that stain positively with each SCT-EGFP were shown. The data represent the mean ± the SD of triplicate analyses. (B) One week after the first or second stimulation, splenocytes were purified and then stimulated with 10 μM of Tax180-188 or NLEnv371-379 peptides for 48 hours. Production of IFN-γ in the culture supernatants was measured by ELISA. The data represent the mean ± the SD of triplicate wells.

By using SCTs fused with EGFP, we have tried to detect Tax-specific CTLs in rats infected with HTLV-I. To prepare HTLV-I infected rats, we have intraperitoneally inoculated F344 rats with 1 × 10^7 ^FPM1.BP cells 3 times. One week after the last inoculation, splenocytes were purified and subjected to FACS analysis to detect Tax-specific CTLs. At first, we tried to detect the CTLs in unstimulated splenocytes, but have so far failed in the attempt, probably because of the low frequency of Tax180-188-specific CTLs in HTLV-I infected rats prepared in this study (data not shown). Thus, we have stimulated the splenocytes in vitro with formalin-fixed FPM1.BP cells twice with 1-week intervals and examined the frequency of Tax180-188-specific CTLs 1 week after each stimulation. As shown in Figure 6A, SCT-EGFP staining of splenocytes from an HTLV-I-infected rat revealed that 11.0 ± 6.5% of CD8+ cells were specifically bound to the SCT-EGFP with Tax180 after the first stimulation. Also, the second stimulation of splenocytes with FPM1.BP expanded the RT1.AlSCTaxL-EGFP positive cell population to 16.5 ± 1.4% of CD8+ cells. In contrast, we were unable to detect a significant level of Tax180-188 CTL induction in the splenocytes derived from a PBS-inoculated uninfected rat. The SCT-EGFP with NLEnv371 did not bind to CD8+ cells derived from an HTLV-I-infected or uninfected control rat. To assess the comparability of the SCT-EGFP staining to other antigen-specific T-cell screening systems, we have stimulated the splenocytes with Tax180-188 or NLEnv371-379 peptides and examined IFN-γ production in the culture. As shown in Figure 6B, stimulation with Tax180-188 peptides induced a significant level of IFN-γ production by splenocytes from an HTLV-I-infected rat after first stimulation. In splenocytes that received the second stimulation, we have detected the enhanced production of IFN-γ after addition of Tax180-188 peptides in an HTLV-I-infected rat, but not in an uninfected control rat. This induction of IFN-γ production was specific to Tax180-188 peptides, because NLEnv371-379 peptides failed to induce a significant level of the cytokine production. Thus, these results indicated that RT1.AlSCTaxL-EGFP fusion proteins were able to detect Tax180-188 specific CTLs in primary splenocytes derived from an HTLV-I-infected rat and that the detection of the epitope specific CTLs by SCT-EGFP fusion proteins was comparable to the assessment of epitope specific production of IFN-γ.

## Discussion

In this study, by using epitope expressing SCTs of rat MHC Class I, we have developed an activation and detection system of HTLV-I Tax-specific CTLs which can be applicable for analyzing CTL responses in a rat model system of HTLV-I infection. The SCT system has been developed in mouse and human MHC-I with its corresponding epitopes [25,26], but not in rat MHC-I. Based on the information previously reported on the mouse system [21], we have designed expression vectors for SCTs of rat RT1.A^l ^and successfully obtained the constructs which can activate epitope specific CTLs in vitro. We have further developed the CTL detection system by combining the SCT complex with EGFP, which should be transferred to epitope specific CTLs as previously reported [23]. Because of the poor availability of MHC-I tetramers in rats, development of this system will provide various benefits in analyzing the role of CTLs in a variety of disease models in rats.

The Tax180-188 epitope used in this study was previously identified by epitope mapping analysis and was actually confirmed to be one of the major epitopes presented by RT1.A^l ^in F344 rats infected with HTLV-I or immunized with Tax protein [20,27]. On the other hand, NLEnv371-379 epitope was predicted by "SYFPEITHI epitope prediction algorithm" [24] and was given 27 points in the scoring system. Since Tax180-188 was given the same points as NLEnv371-379 scored, it would be reasonable to assume that NLEnv371-379 epitope was equivalently presented by RT1.A^l ^in our present experiments. Nevertheless, only SCTs with Tax180, but not with NLEnv371 can recognize and activate Tax180-188 specific CTLs. Moreover, SCTs with Tax180 did not recognize another CD8+ T cell line, G14, which is not specific to Tax180-188. These results indicated that the SCTs of RT1.A^l ^engineered in the present study appropriately presented the Tax epitope to the corresponding CTLs. However, it is still necessary to establish new CTL lines with different epitope specificities for further confirming the epitope specificity of SCTs used in this study. In addition, it is important to identify new CTL epitopes in rat model of HTLV-I infection for better understanding of the relationship between diversity of HTLV-I-specific CTLs and the virus-related diseases. Especially, recently identified HTLV-I basic leucine zipper factor (HBZ) is the most important factor to be analyzed as a CTL target because of its possible involvement in ATL development [28]. The SCT system together with RT1.A^l^-EGFP complex should be applicable for the search of new epitopes in F344 rats. Indeed, Tomaru et. al successfully detected new CD8+ T cell epitope from the envelope region of HTLV-I using HLA-A2-EGFP fusion proteins [23].

MOLT-4/RT1AlSCTax180L cells induced the production of IFN-γ by 4O1/C8 CTLs. However, the activated 4O1/C8 cells failed to proliferate, but rather tended to decrease the number in the mixed culture (Figure 3). This is in dramatic contrast to the results observed in the mixed culture of 4O1/C8 with FPM1.BP, wherein both IFN-γ production and cell proliferation were enhanced. Although the exact mechanism of this difference is not clear, our results suggest that the failure of CTL expansion was due to the enhanced apoptosis induced by RT1AlSCTax180L. In this regard, it has been reported that the presence of CD4+ helper T cells reduced CTL susceptibility to AICD through a cell contact-dependent mechanism [29]. Thus, it is possible that activation of 4O/1C8 by RT1AlSCTax180L may fail to induce the protective signal from AICD in the CTLs. Since a syngeneic CD4+ T cell line, FPM1.BP, was able to induce the expansion of 4O1/C8 in spite of the apparent AICD induction, it is also possible that MOLT-4/RT1AlSCTax180L cells failed to trigger the signal(s) which 4O1/C8 was able to activate for the induction of CTL proliferation. Actually, as shown in Figure 3D, we have detected the enhanced production of IL-2 in the mixed culture of 4O1/C8 with FPM1.BP, suggesting the involvement of the IL-2 signal transduction pathway in the proliferation of 4O1/C8. Further analysis is required to clarify the activation mechanisms of CTLs in the rat system for inducing better immune response by SCTs. Nevertheless, it may be still possible to apply the pEF/RT1AlSCTax180L vector for inducing Tax-specific CTL response in rats, since similar SCT complex expressing human papillomavirus-16 E6 antigen was shown to induce protective immunity against the virus in a mouse system in vivo [30]. Thus, it will also be necessary to assess the in vivo effect of the rat SCTs for the evaluation of the system as a therapeutic tool in HTLV-I infection.

Previous reports suggested that insufficient T cell response against HTLV-I is a potential risk factor for ATL. Among HTLV-I infected individuals, the infrequency of HTLV-I-specific CTL induction in vitro has been reported in ATL patients [12,13,31]. Moreover, a recent study using various tetramers clearly demonstrated the reduction of the frequency and diversity of anti-Tax CTLs in ATL patients [32]. The importance of HTLV-I-specific T cell immunity in anti-tumor surveillance was also supported by a previous report showing that Tax-specific CTL response was strongly activated in ATL patients who obtained complete remission after HSCT [16]. These observations suggested the importance of Tax-specific CTLs for prevention and therapy of ATL and should be further verified using suitable animal models. Rats have been used for a number of studies on HTLV-I infection, because they are susceptible to the virus and because the virus-transformed T cell lines can be established in vitro [33,34]. It has previously shown in a rat model that HTLV-I specific T cells were important to inhibit the growth of virus-infected cells in vivo [17]. Moreover, the association of elevated proviral load with insufficient T cell immunity has been also observed in a rat model of oral HTLV-I infection [35]. In this model, it has further demonstrated that re-immunization of orally HTLV-I-infected rats resulted in a reduction of the proviral load [36]. Although these results further support the importance of Tax-specific CTLs for the prophylaxis and treatment of ATL, detailed analysis to understand the interplay between epitope-specific CTLs and HTLV-I infected cells in vivo has not been performed yet. This is mainly due to the lack of tools to identify epitope specific CTLs in rats. In this study, we have demonstrated that the SCT-EGFP system was able to detect Tax180-188 specific CTLs in splenocytes derived from an HTLV-I-infected rat and that the detection of the epitope-specific CTLs by SCT-EGFP system was comparable to the measurement of peptide-induced IFN-γ production. Thus, the activation and detection system established in this study should be useful for further verifying the strategies to fight against HTLV-I.

## Conclusion

In this study, we have generated a SCT of rat MHC-I linked to Tax epitope peptide, which can be applicable for the induction of Tax-specific CTLs in rat model systems. We have also established a detection system of Tax-specific CTLs by using cells expressing SCTs fused with EGFP. These systems will be useful tools in understanding the role of HTLV-I specific CTLs in HTLV-I pathogenesis.

## Methods

### Cell lines

An HTLV-I-immortalized cell line, FPM1.BP, was established previously from an F344/N Jcl-rnu/+ rat [37]. The cells were maintained in RPMI 1640 with 10% heat-inactivated FCS (Biosource, Rockville, MD), penicillin, and streptomycin. A CD8+ Tax-specific CTL line, 4O1/C8, and an IL-2-dependent HTLV-I-negative CD8+ cell line, G14, were also established previously from F344/N Jcl-rnu/+ rats [19]. These cells were maintained in RPMI 1640 medium with 10% FCS and 20 U/ml of IL-2 (PEPROTECH, London, UK). For the maintenance of 4O1/C8 cells, periodical stimulation with formalin-fixed FPM1.BP cells is also required, because their growth is dependent on RT1.A^l^-restricted presentation of Tax180-188 epitope [37]. Human 293T cells were maintained in Dulbecco's modified Eagle's medium supplemented with 10% FCS and MOLT-4 cells were cultured in RPMI 1640 medium with 10% FCS.

### Plasmid DNA construction

Plasmid constructs were generated using standard techniques and were confirmed DNA sequence analysis. Briefly, Rat MHC-I (RT1.A^l^) and β_2_m cDNAs were amplified by PCR using G14 cell-derived cDNAs as templates. The PCR products of RT1.A^l ^and rat β_2_m were cloned to the pCR2.1 vector using TA cloning kit (Invitrogen, Carlsbad, CA) and were designated as pCR2/RT1Al and pCR2/rβ_2_M, respectively. The DNA encoding the epitope peptide-β_2_m-RT1.A^l ^fusion protein was synthesized by a multistep PCR using pCR2/rβ_2_M as a template. The first PCR was performed to add the L2 sequence at the 3' end of β_2_m gene. The following 2 or 3 steps of PCRs were performed to add NotI site-containing region of RT1.A^l ^signal sequence and the epitope sequence fused with the L1 linker at the 5' end of β_2_m gene and the AvaI site-containing region of RT1.A^l ^α_1 _domain at the 3' end. The primers used for these stepwise reactions were summarized in Table 1. The third or forth PCR product was digested with NotI and AvaI, and then cloned between NotI and AvaI sites of pCR2/RT1Al to construct pCR2 vectors containing peptide-β_2_m-RT1.A^l ^fusion sequence. The obtained constructs were further amplified by PCR to add BamH1 and Bsp1407I sites at the 5' and 3' end of the fusion constructs, respectively and were ligated between BamH1 and Bsp1407I sites of pEFGFP vector [27]. In this study, we constructed 4 expression vectors with 2 different epitopes and 2 different lengths of linkers. The diagram of SCT expression vectors established in this study was shown in Figure 1B. The short or long linkers consist of 10 or 15 residues of L1 and 15 or 20 residues of L2, respectively. Tax180-188 epitope was previously identified by epitope mapping in a Tax-specific CTL line [20]. A putative epitope in the envelope of HIV-1 NL4-3 strain, NLEnv371-379 was determined by epitope prediction data via [24]. We also constructed the pEF/RT1Al plasmid, which expresses RT1.A^l ^protein.

**Table 1:** Primers to construct SCTs of RT1.A^1^

**Constructs**		**First PCR**	Second PCR*	Third PCR*	**Forth PCR**
**RT1AlSCTax180S**	Sense primer	ATTCAGAAAACTC CCCAAATTCAAG TGTAC	GGCCGCCCTGGCCCCGACCCAGACC CGCGCG**GGGGCCTTCCTCACCAATG** **TTCCCTAC**GGAGGTGGCGGGTCCGG AGGTGGCGGGTCCATTCAGAAAACT CCCCAAATTCAAGTGTACTCTCGCC ATCCA	CCTGCTGCTGGCGGCC GCCCTGGCC CCGAC	Not applicable
	
	Reverse primer	CGCCACCTCCCAT GTCTCGGTCCCA GGTGA	CCGAGGCCGGGCCGGGACACGGCGA TGTCGAAATACCGCATCGAGTGAGA GCCGGACCCGCCACCTCCGGACCCG CCACCTCCGGACCCGCCACCTCCCA TGTCTC	CCGGGGCTCCCCGAG GCCGGGCCGGGACAC GGCGATGTC	Not applicable

**RT1AlSCNLEnv371S**	Sense primer	ATTCAGAAAACTC CCCAAATTCAAG TGTAC	GGCCGCCCTGGCCCCGACCCAGACC CGCGCG**CACAGTTTTAATTGTGGAG** **GGGAATTT**GGAGGTGGCGGGTCCGG AGGTGGCGGGTCCATTCAGAAAACT CCCCAAATTCAAGTGTACTCTCGCC ATCCA	CCTGCTGCTGGCGGCC GCCCTGGCCCCGAC	Not applicable
	
	Reverse primer	CGCCACCTCCCAT GTCTCGGTCCCA GGTGA	CCGAGGCCGGGCCGGGACACGGCGA TGTCGAAATACCGCATCGAGTGAGA GCCGGACCCGCCACCTCCGGACCCG CCACCTCCGGACCCGCCACCTCCCA TGTCTC	CCGGGGCTCCCCGAG GCCGGGCCGGGACAC GGCGATGTC	Not applicable

**RT1AlSCTax180L**	Sense primer	ATTCAGAAAACTC CCCAAATTCAAG TGTAC	GGAGGTGGCGGGTCCATTCAGAAAA CTCCCCAAATTCAAG	GGCCGCCCTGGCCCCG ACCCAGACCCGCGCG **GGGGCCTTCCTCACC** **AATGTTCCCTAC**GGA GGTGGCGGGTCCGGA GGTGGCGGGTCCGGA GGTGGCGGGTCC	CCTGCTGCTGGCGGC CGCCCTGGCCCCGAC
	
	Reverse Primer	CGCCACCTCCCAT GTCTCGGTCCCA GGTGA	GGCCGGGACACGGCGATGTCGAAAT ACCGCATCGAGTGAGAGCCGGACCC GCCACCTCCGGACCCGCCACCTCCG GACCCGCCACCTCCGGACCCGCCAC CTCCCATGTCTC	CCGGGGCTCCCCGAG GCCGGGCCGGGACAC GGCGATGTC	GCTCCCCGAGGCCGG GCCGGGACACGGCGA

**RT1AlSCNLEnv371L**	Sense Primer	ATTCAGAAAACTC CCCAAATTCAAG TGTAC	GGAGGTGGCGGGTCCATTCAGAAAA CTCCCCAAATTCAAG	GGCCGCCCTGGCCCCG ACCCAGACCCGCGCG **CACAGTTTTAATTGT** **GGAGGGGAATTT**GGA GGTGGCGGGTCCGGA GGTGGCGGGTCCGGA GGTGGCGGGTCC	CCTGCTGCTGGCGGC CGCCCTGGCCCCGAC
	
	Reverse Primer	CGCCACCTCCCAT GTCTCGGTCCCA GGTGA	GGCCGGGACACGGCGATGTCGAAAT ACCGCATCGAGTGAGAGCCGGACCC GCCACCTCCGGACCCGCCACCTCCG GACCCGCCACCTCCGGACCCGCCAC CTCCCATGTCTC	CCGGGGCTCCCCGAG GCCGGGCCGG GACACGGCGATGTC	GCTCCCCGAGGCCGG GCCGGGACACGGCGA

* Bold cases indicate sequences encoding Tax180-188 or NLEnv371-379 epitope peptide.

For the generation of SCT-EGFP expression vectors, RT1AlSCTax180L or RT1AlSCNLEnv371L cDNAs were further amplified by PCR to delete a stop codon and to add KpnI and BamHI sites at the 5'- and 3'- termini, respectively. The pEF/RT1AlSCTax180L-EGFP and pEF/RT1AlSCNLEnv371L-EGFP vectors were generated by insertion of the corresponding PCR products between KpnI and BamHI sites of pEFGFP vector.

To confirm the accuracy of vectors used in this study, all established constructs were subjected to sequence analysis using ABI PRISM 310 Genetic Analyzer (Applied Biosystems, Foster City, CA) according to the manufacturer's instruction.

### Cytokine production assay

An HTLV-I Tax-specific CTL line, 4O1/C8 (2 × 10^5^/well), was mixed with various stimulator cells (2 × 10^5^/well). In some experiments, stimulator cells were fixed with 1% formalin in PBS or pulsed with 10 μM of Tax180-188 or NLEnv371-379 peptide (MBL, Nagoya, Japan) before incubation with the CTL. For the stimulation of primary splenocytes with peptides, 5 × 10^4 ^of splenocytes were incubated with 10 μM of Tax180-188 or NLEnv371-379 peptides. After the indicated period of mixed culture, supernatants were harvested and were subjected to rat IFN-γ (eBioscience Inc., San Diego, CA), TNF-α ELISA (eBioscience Inc.), or IL-2 (R&D Systems Inc., Minneapolis, MN) in accordance with the manufacturer's instructions.

### Flow cytometric analysis

For the assessment of SCT expression, MOLT-4 cells transfected with various SCT expression vectors were stained with an anti-rat MHC-I antibody (BD Bioscience, San Jose, CA) for 30 min on ice, washed three times with 1% FCS in PBS, and then stained with FITC-conjugated goat anti-mouse IgG+IgM. After being washed, the cells were fixed with 1% formalin in PBS prior to analysis on a FACScalibur (BD Bioscience). For the detection of Tax180-188 specific CTLs by the mixed culture with SCT-EGFP expressing cells, 4O1/C8 or splenocytes were incubated with 293T cells expressing SCT-EGFP fusion proteins for 1 hour. Cells in the mixed cultures were stained with phycoerythrin (PE)-conjugated anti-rat CD8 (clone OX-8; BD Bioscience) for 30 min on ice, washed three times with 1% FCS in PBS, fixed with 1% formalin in PBS, and then subjected to FACS analysis.

### Cell growth assay

FPM1.BP or MOLT-4 cells with SCTs were fixed with 1% formalin in PBS for 20 min and then washed four times with RPMI 1640 medium. These formalin-fixed cells (1 × 10^5^/well) were incubated with 4O1/C8 (1 × 10^5^/well) in each well of 96-well round-bottom microtiter plates for 3 days at 37°C. The number of growing cells was determined by using a Cell Counting Kit-8 (Dojinndo Laboratories, Kumamoto, Japan) in accordance with the manufacturer's instructions.

### Apoptosis analysis

Formalin-fixed MOLT-4 or FPM1.BP cells (1 × 10^5^/well) were incubated with 4O1/C8 (1 × 10^5^/well) in each well of 96-well round-bottom microtiter plates for 24 hours at 37°C. The percentage of 4O1/C8 cells undergoing apoptosis was determined by FACS analysis using the Annexin V-FITC Apoptosis Detection Kit (MBL) in combination with a PE-conjugated anti-rat CD8 antibody (BD Bioscience).

### Immunofluorescence staining

SCT-EGFP expressing 293T cells were cultured with 4O1/C8 in each well of 96-well round-bottom microtiter plates for 1 hour at 37°C. Cells in the mixed cultures were attached on slide glasses (Matsunami Glass Ind., Japan) by centrifugation, fixed with 4% paraformaldehyde in PBS and then stained with an anti-rat CD8 antibody (BD Bioscience) in combination with Cy3-conjugated goat anti-mouse IgG (H+L) (Jackson ImmunoResearch Laboratories, West Grove, PA). Images were examined with a confocal microscope system (FluoView; Olympus, Tokyo, Japan).

### Preparation of immune splenocytes

Female F344/Jcl rats were purchased from Clea Japan, Inc. (Tokyo, Japan). Four-week-old F344/Jcl rats were intraperitoneally inoculated with 1 × 10^7 ^FPM1.BP cells. The rats received two boost inoculations with the same dose at 2 and 10 weeks after initial inoculation. One week after the last inoculation, splenocytes were isolated, purified by centrifugation on a density separation medium (Lympholyte-Rat; Cedarlane, Ontario, Canada) and subjected to the analysis for the detection of Tax180-188-specific CTLs or the quantification of IFN-γ production. All rats were maintained at the P3 level animal facilities in Laboratory of Animal Experiment, Institute for Genetic Medicine, Hokkaido University. The experimental protocol was approved by the Animal Ethics Review Committee of our University.

### Statistical analysis

Comparisons between individual data points were made using a Student's *t*-test. Two-sided P values < 0.05 were considered statistically significant.

## Competing interests

The authors declare that they have no competing interests.

## Authors' contributions

TO designed the study, performed all the experiments and the analysis, and wrote the manuscript. MN performed microscopic examinations. HO made contributions to design the study and participated in flow cytometric analysis. RT participated in flow cytometric analysis and HTLV-I infection experiments. HS made contributions to design the study and drafted the manuscript.
